# Effect of Montmorillonite on 4-Nonylphenol Enrichment in Zebrafish

**DOI:** 10.3390/ijerph15061217

**Published:** 2018-06-10

**Authors:** Kun Qian, Xiaofeng Jiang, Laiyu Sun, Guoqing Zhou, Haixia Ge, Xinqiang Fang, Li Xiao, Qiong Wu

**Affiliations:** 1 School of Life Science, Huzhou University, Huzhou 313000, China; sunly@zjhu.edu.cn (L.S.); zgq@zjhu.edu.cn (G.Z.); gehaixia@zjhu.edu.cn (H.G.); fxq@zjhu.edu.cn (X.F.); tdxiao427@zjhu.edu.cn (L.X.); 17855124660@126.com (Q.W.); 2 College of Life Science, Zhejiang Sci-Tech University, Hangzhou 310018, China

**Keywords:** 4-nonylphenol, montmorillonite, zebrafish, enrichment, enzyme activity

## Abstract

The aim of this study was to investigate the effect of montmorillonite on nonylphenol (4-nonylphenol, 4-NP) enrichment in a zebrafish model. The AB strain zebrafish were used as the animal subjects, and three concentration gradients were set for both nonylphenol and montmorillonite, according to their actual concentrations in aquaculture water in Huzhou City. A group treated with nonylphenol alone was also set, adding up to 12 experimental groups. Concentrations of nonylphenol enriched in the liver, muscle and gills of zebrafish were detected by solid phase microextraction–high performance liquid chromatography at Days 7, 15 and 30, respectively. Additionally, the relative enzymatic activity of superoxide dismutase (SOD) and the glutathione S-transferase (GST) were also detected, and the data were statistically analyzed. The results showed that the concentrations of nonylphenol in zebrafish peaked at Day 7 and gradually decreased afterwards for all the experimental groups. The montmorillonite reduces short-term accumulation of nonylphenol in gills, and the high concentration of nonylphenol facilitates its enrichment in liver and muscle, while the low concentration of nonylphenol does not. Meanwhile, the low concentration of nonylphenol in liver exerts an influence on the inductive effect of SOD and GST, while the high concentration of nonylphenol shows the inhibiting effect of SOD and GST.

## 1. Introduction

Environmental endocrine-disrupting compounds (EDCs) interfere with the synthesis, release, transport, metabolism, binding, action or elimination of endogenous hormones and then impact the normal endocrine system of organisms, leading to reproduction and immune dysfunctions [1]. In addition to the reversible or irreversible biological effects on the organisms, the offspring or the population, EDCs also compromise the disease resistance of the body [2,3] and even cause diseases and cancer [4,5,6,7]. For instance, nonylphenol (NP), a common industrial raw material, is a typical phenolic environmental hormone and mainly accumulates in water bodies with a solubility of 5.43 mg/L [8]. NP is a general term for the isomers, and its formula is C_6_H_4_(OH)C_9_H_19_. 4-NP is one of the major components of NP, almost 90% of the total. This chemical presents genotoxicity, developmental toxicity, immunotoxicity and neurotoxicity [9,10,11,12,13]. Additionally, it may deposit in living organisms and exhibit biological effects via the water body, as well as through the food chain, and the effect of environmental EDCs might be more harmful after enrichment by the food chain [14,15].

The dose of environmental EDCs is generally low in nature, and the correlation between their effect and dose is complex. For example, the toxicity of bisphenol A is stronger at low doses than at high doses [16,17]. The application of biomarkers is a common method to evaluate and analyze the toxic effects of toxicants. The antioxidant enzymes of zebrafish are commonly-used biomarkers [18,19,20]. However, the dose used in the current study of the dose-effect relationship is basically the dose of toxicants’ exposure in the environment, and the study of the concentration-effect relationship between toxicant concentrations and markers in tissues or organs of zebrafish has rarely been reported. The situation is more complicated in an actual natural environment, where a variety of substances, especially some nanoparticles in the water, modify the biological effects of environmental EDCs and impact the adsorption, transport, enrichment and even the toxicity of EDCs [21,22,23]. Montmorillonite (MMT) is a typical layered aluminosilicate mineral that is adsorptive, hydrophilic, electrically charged, dispersedly suspended and swells in water [24,25,26]; therefore, it is widely used in medicine, aquaculture and sewage treatment [27,28,29,30,31,32]. MMT, as a common nanoparticle in water bodies, has the potential to enhance the toxicity of harmful substances and meanwhile reduces the accumulation of harmful substances and exhibits a detoxification function in aquatic animals [33,34,35,36]. Few studies have reported the role of MMT in a specific water environment, and in theory, MMT could affect aquatic animals, resulting in them absorbing NP due to the adsorptivity of MMT; while the NP enters the body, MMT could also affect the transport and metabolism of NP. These effects can be found through the distribution patterns of in vivo NP affected by MMT; however, there is still no research on these effects that has been reported. In the present study, the effect of MMT on NP accumulation in zebrafish was investigated in a water environment using NP as a specific toxic substance. In addition, the relationship between the concentration of NP in liver and the enzyme activity of SOD and GST was also analyzed.

## 2. Materials and Methods

### 2.1. Instruments and Experimental Materials

HPLC (high performance liquid chromatograph, LC-20AT, Shimadzu Corporation. Shanghai, China) and solid phase microextraction (Supelco, 75 μm PDMS/DVB) were performed. Zebrafish (*Danio rerio*) AB strain (purchased from a local fish market), 3–4 months of age, both sexes, weighing approximately 1.5–2 g and having a body length of 2.5–3.5 cm, were kept in recirculating water at 28 °C under standard laboratory conditions for two weeks. 4-Nonylphenol (CAS: 104-40-5), analytically pure, 98%, was purchased from the Shanghai Ziyi Reagent Company (Shanghai, China). The pharmaceutical-grade montmorillonite (MMT) was purchased from Gaoyu Bentonite Company (Anji, China). The SOD and GST Assay Kits were purchased from the Jiancheng Bioengineering Institute (Nanjing, China).

All animal care and experimental procedures were approved by the Committee on Animal Care and Use and the Committee on the Ethics of Animal Experiments of Huzhou University and Zhejiang Sci-Tech University. All methods were performed in accordance with the relevant guidelines and regulations.

### 2.2. Experimental Methods

#### 2.2.1. HPLC Parameter Settings

Chromatographic column: Waters Symmetry C18 (4.6 × 150 mm, 5 μm); mobile phase: methyl alcohol: H_2_O = 26:74; detection wavelength: 225 nm; flow velocity: 1.0 mL·min^−1^; the column temperature was at 35 °C; inlet sample quantity: 20 μL.

#### 2.2.2. The Methodology of 4-NP Detection Based on the HPLC Method

(1) Accuracy: Taking six parallel samples of 4-NP with the identical concentration, the concentration of each sample was 2.092 × 10^3^ μg/L according to the HPLC detection. The RSD (relative standard deviation) was also calculated.
(2) Confirmation of the quantitation limit (LOQ) and detection limit (LOD): The standard 4-NP samples were diluted, then the LOQ and LOD were set as S/N = 10:1 and S/N = 3:1, respectively.
(3) The recoveries of 4-NP: The zebrafish tissue samples of liver, muscle and gills, as well as the water sample were added to the final concentration of 4-NP at 2.092 × 10^1^, 2.092 × 10^2^ and 2.092 × 10^3^ μg/L, respectively. The water sample was processed in accordance with Section 2.2.3, and the zebrafish tissue samples were processed in accordance with Section 2.2.5. The processed samples were analyzed by HPLC, and the recoveries of 4-NP were acquired.
(4) Standard curve: The zebrafish tissue samples of liver, muscle and gill, as well as water samples, were added to the final concentration of 4-NP at 2.092, 2.092 × 5^1^, 2.092 × 5^2^, 2.092 × 5^3^, 2.092 × 5^4^ and 1.046 × 5^5^ μg/L, respectively. The water sample was processed in accordance with Section 2.2.3 (under the optimum condition), and the zebrafish tissue samples were processed in accordance with Section 2.2.5. The processed samples were analyzed by HPLC, and the absorption peak areas were measured. Next, the linear equation between the concentration and the absorbance of 4-NP was established.

#### 2.2.3. The Conditions of Solid Phase Microextraction

The water samples derived from aquaculture water were filtered by a microfiltration membrane (0.45 μm), and the assembly of the adsorption time (60, 40, 30 and 20 min) of SPME and the resolution time (40, 30, 20, 10, 9, 7, 5 and 3 min) of SPME can confirm the optimal adsorptional analytical conditions through HPLC analysis. The experimental procedure of SPME was in accordance with the instructions.

#### 2.2.4. Exposure Measurement and Grouping

(1) Determination of the exposure concentration of 4-NP:

A total of 10 typical aquaculture water samples in the Huzhou area were selected, with the average concentration of 4-NP detected by high performance liquid chromatography regarded as a 1× exposure concentration of 4-NP.

(2) Determination of MMT concentration:

The accumulation in seven consecutive days was calculated as a 1× exposure concentration of MMT on the basis that the depth of the aquaculture water system was 1.2–1.7 m. The annual input of commercial feed per mu was 350–500 kg, and 2–5 kg of MMT in aquatic feed per ton were added. The result was 2.949 × 10^−5^ g/L. 

(3) Exposure test grouping:

The samples were divided into 17 experimental groups, respectively, 1×, 10× and 100× 4-NP exposure groups, 1/100, 1× and 100× MMT exposure groups, nine pairwise combinations between 1/100, 1× and 100× MMT exposure concentrations and 1×, 10× and 100× 4-NP exposure concentrations, the organic solvent group (with 1 mL ethanol added) and the test water group, with three parallel tests in each group. 4-NP with different amounts in the experimental groups was dissolved with 1 mL ethanol. The solid MMT was dissolved with some deionized water first, then added into deionized water along with the 4-NP alcoholic solution.

#### 2.2.5. The Treatment of Zebrafish Tissue Samples

Each of the 25 zebrafish were raised in 20 L of aquaculture water (the aquaculture water was prepared by deionized water with different concentrations of 4-NP and MMT) in a tank, with a pH of 7.0 ± 0.5 (adjusted by NaHCO_3_). A fluorescent lamp was chosen to simulate natural light, replacing half of the aquaculture water every 24 h. The zebrafish were fed with the commercial feeds (without MMT), and the fish maintenance and the feeding protocol have been described by Lee et al. [37]. After being raised for 7, 15 and 30 days, six fish were randomly selected from each tank, respectively. The tissue samples of liver, muscle and gills were extracted and stored at −20 °C. 

Tissue samples derived from two fish (sex chosen at random) were classified into one group. The tissue homogenates were added to 10 mM/L HCl up to 9 mL, stored at 4 °C for 24 h, then each group was centrifuged for 10 min (6000 rpm at 4 °C). The supernatant was filtered with a 0.45-μm filter membrane and was diluted by ultrapure water to 15 mL. Then, the diluent was processed in accordance with Section 2.2.3 (under the optimum condition).

#### 2.2.6. Determination of the Concentrations of 4-NP in Tissues and Data Analysis

The concentrations of 4-NP in treated samples were detected by HPLC in accordance with Section 2.2.1. Statistical evaluations of the significant differences among the means of experimental groups were performed using Student’s *t*-test (MS Excel 2010). 

#### 2.2.7. Measurements of Enzymatic Activity

The liver samples were derived from 2 fish of each experimental group, and the sampling and the enzymatic activity determinations of SOD and GST were in accordance with the kit instructions.

## 3. Results

### 3.1. Parameters of 4-NP Testing Methodology

#### 3.1.1. The Essential Parameters 

The LOQ was set at a 4-NP concentration of 1.046 μg/L, while the LOD was set at a 4-NP concentration of 0.4184 μg/L. The accuracy was set at a 4-NP concentration of 2.092 × 10^3^ μg/L with the RSD 2.25% (*n* = 6). The recovery rate of 4-NP ranged from 77.797%–89.274% (Table 1).

#### 3.1.2. Standard Curve

The peak area of the elution curve of 4-NP from the samples derived from zebrafish tissues (liver, muscle and gills) and aquaculture water have been measured, and the binary linear regression equation has been established by contrasting the peak area of each sample with the 4-NP content of each sample (Table 2). The correlation coefficients of these two factors are also listed in Table 2. 

### 3.2. Optimum Condition for SPME

Extraction was processed by 75 μm of PDMS/DVB. The optimal adsorption time was 20 min, and the resolution time was 5 min.

### 3.3. Exposure Dose of 4-NP

The mean 4-NP contents of 10 measured water samples was 3.2133 μg/L, while the exposure doses of 4-NP were 3.2133 μg/L, 32.133 μg/L and 321.33 μg/L, respectively.

### 3.4. The Determination Results and Data Analysis of 4-NP in Tissues 

The 4-NP contents in liver, muscle and gills of zebrafish at Day 7, Day 15 and Day 30 were measured and calculated for single factor analysis of variance with Excel. The results showed that 1/100, 1× and 100× MMT exposure concentrations, organic solvent ethanol and test water had no effect on the experimental results (in the following tables, N1, N2 and N3 respectively represent low, medium and high concentrations of 4-NP, and M1, M2 and M3 respectively represent low, medium and high concentrations of MMT).

#### 3.4.1. Variance Analysis of 4-NP Contents in Liver 

**Figure 1:**
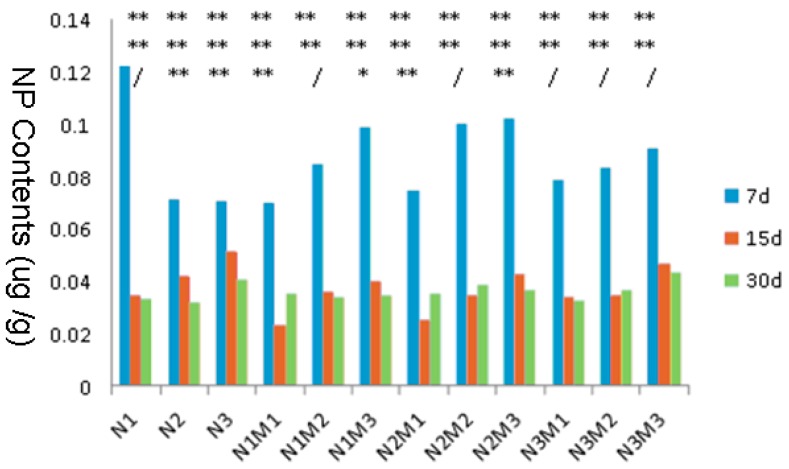
Statistical analysis of the content variation of 4-nonylphenol (4-NP) in liver at Day 7, Day 15 and Day 30. N, 4-NP; M, montmorillonite.

Table 3, Table 4 and Table 5 represent the significance analysis of the mean difference of the 4-NP contents in liver at Day 7, Day 15 and Day 30 between the groups (N1, N2 and N3 respectively represent low, medium and high contents of 4-NP, and M1, M2 and M3 respectively represent low, medium and high concentrations of MMT. In addition to the row of 4-NP content, other columns with the asterisk (*) represents the *p*-value, where “/” represents *p* ≥ 0.05, * represents *p* < 0.05, ** represents *p* < 0.01 and blank represents no comparison.). The changes of 4-NP contents of each experimental group at Day 7, Day 15 and Day 30 are shown in Figure 1 (* and “/” at the top of the figure: the first row represents the analysis of the difference of significance between 4-NP contents at Day 7 and Day 15, the second row represents analysis at 7 d and 30 d, while the third row represents analysis at Day 15 and Day 30). The data analysis showed that whether with or without MMT, the concentration of 4-NP in liver has been on the decline over time. On the seventh day, when MMT was non-existent, the concentration of 4-NP in liver was highly enriched with a low dosage, but there was no such effect at Day 15 and Day 30. By contrast, 4-NP cannot be enriched in liver at any time while MMT exists.

#### 3.4.2. Variance Analysis of 4-NP Contents in Muscle

**Figure 2:**
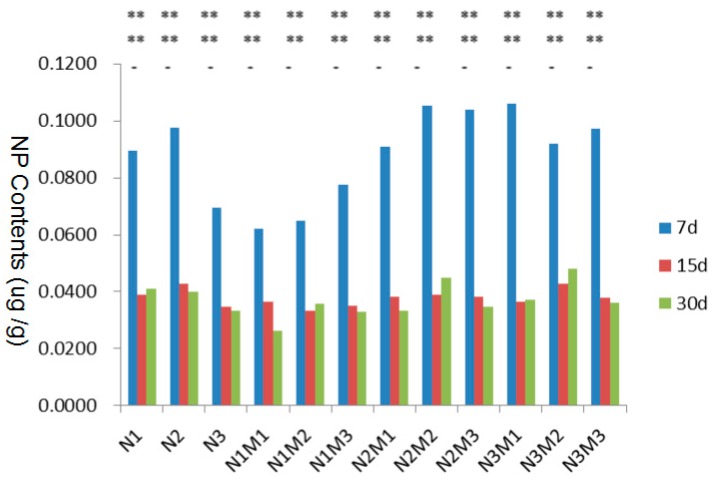
Statistical analysis of the content variation of 4-NP in muscle at Day 7, Day 15 and Day 30.

The 4-NP contents in zebrafish muscle at Day 7, Day 15 and Day 30 are listed in Table 6, Table 7 and Table 8, respectively. Significance analysis of the means of all experimental groups is also demonstrated in these tables. The content variation of 4-NP in every experimental group at Day 7, Day 15 and Day 30 is illustrated in Figure 2. The data analysis shows that whether with or without MMT, the concentration of 4-NP in muscle has been on the decline over time. On the seventh day, when MMT was nonexistent, the concentration of 4-NP in muscle was highly enriched with a low and a medium dosage, and this effect could last for up to 15 days. By contrast, 4-NP cannot be enriched in muscle at any time while MMT exists.

#### 3.4.3. Variance Analysis of 4-NP Contents in Gill

**Figure 3:**
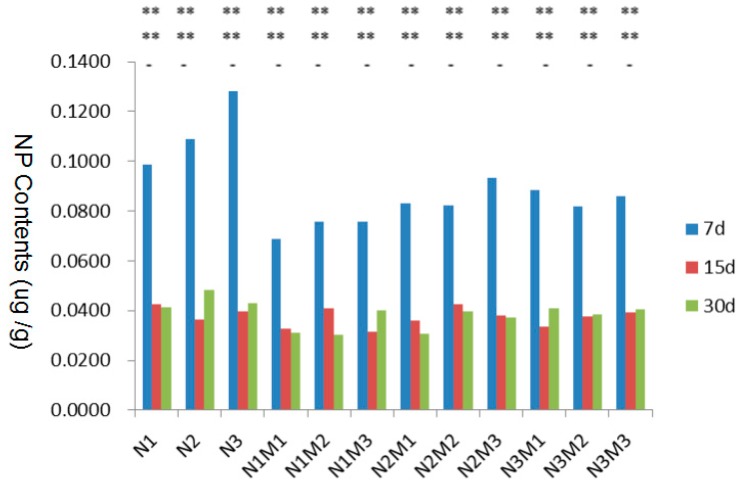
Statistical analysis of the content variation of 4-NP in gill at Day 7, Day 15 and Day 30.

The 4-NP contents in zebrafish gill at Day 7, Day 15 and Day 30 and the significance analysis of the means of all experimental groups are demonstrated in Table 9, Table 10 and Table 11, respectively. The content variation of 4-NP in every experimental group at Day 7, Day 15 and Day 30 is illustrated in Figure 3. The data analysis showed that the concentration of 4-NP in gill has been on the decline over time. The 4-NP enrichment effect in gills was not related to the concentrations of NP and MMT in aquaculture water.

### 3.5. Enzymatic Activity Determinations

**Figure 4:**
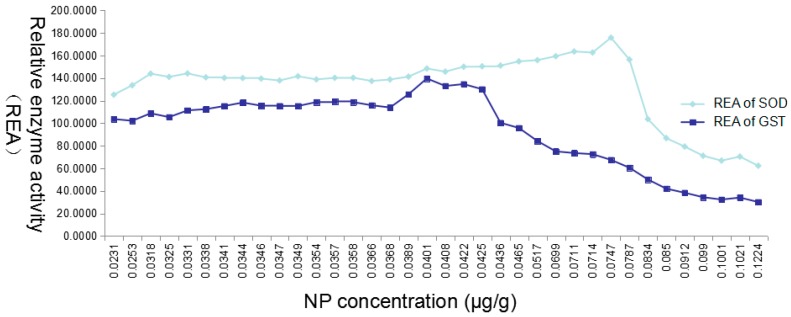
The relative enzyme activity (REA) of SOD and GST under the different 4-NP concentrations.

The average enzymatic activity of SOD and GST of zebrafish within the aquaculture water group, MMT group and organic solvent group in each time slot was detected, and the data were analyzed. The results suggested that the MMT and the organic solvent have no effect on the enzyme activity of SOD and GST (Figure 4). In Figure 4, we set the liver 4-NP as the horizontal axis, and the relative enzymatic activity (the average enzymatic activity of experimental groups/the average enzymatic activity of organic solvent group) as the vertical axis. The results showed that the enzyme activities of SOD and GST were affected by the in vivo 4-NP, and the critical concentrations of 4-NP for the enzyme activities of SOD and GST were 0.0747 μg/g and 0.0401 μg/g, respectively.

## 4. Discussion

With a concentration of 4-NP in a real environment of 3.2133 μg/L, which is three-times that of LOQ, this concentration of 4-NP was set as the minimum concentration of the exposure concentration, and the 4-NP concentrations of 32.133 μg/L and 321.33 μg/L were set as the medium concentration and the maximum concentration. The results suggested that in all experimental groups, the concentration of 4-NP in liver, muscle and gills showed increasing trends at first, and then decreased; the concentration of 4-NP drop in the later stage may be attributed to the zebrafish that had resistance and enhanced the decomposition ability. The concentration of 4-NP in the gills at the same time was not significantly different among groups (*p* > 0.05), suggesting that while 4-NP in aquaculture water was directly in contact with the gills, the concentration of 4-NP in gills was not related to MMT. Generally speaking, MMT in aquaculture water could affect 4-NP enriched in the fish in two ways, and MMT can reduce the concentration of 4-NP in aquaculture water by its adsorption effect. Moreover, MMT in the fish could slow the metabolism of 4-NP and reduce the rate of excretion; and may, in this way, serve the in vivo enrichment of 4-NP. We used the enzyme activity as the indicator to investigate the toxicity effect of 4-NP on zebrafish, and the result showed a complexity of environmental hormones, that is the low dose of 4-NP had a stronger bioactivity than the high dose of 4-NP. In addition, the enzyme activity of SOD was 175.82% of the control, while the enzyme activity of GST was lower (139.65%), suggesting that SOD is more sensitive to 4-NP, and it is also more suitable for the detection index of 4-NP.

The test 4-NP concentration employed in our study was set to 3.2133 μg/L, which was consistent with the environmental 4-NP concentration, and this concentration of 4-NP, as well as the 4-NP concentrations of 32.133 μg/L and 321.33 μg/L were used in the respective experiments. The results showed that the concentrations of 4-NP in the liver, muscle and gills of zebrafish in all the experimental groups reached a peak at Day 7, then decreased at Day 15 and Day 30 (*p* < 0.01). There were no differences in the concentrations of 4-NP in muscle and gills between Day 15 and Day 30 for each group; data from the liver were relatively complicated, i.e., the differences of N2, N3, N1M1, N2M1, N2M3 (*p* < 0.01) and N1M3 (*p* < 0.05) versus the control were significant, while those of the other groups were not. Overall, 4-NP concentrations in the liver, muscle and gill of all the experimental groups were high at first and later decreased, and the declined concentration in the late period could be explained by enhanced resistance or decomposition by the zebrafish. In the absence of MMT, enrichment of 4-NP in the liver at Day 7 was more significant at a lower dose of 4-NP, namely N1 > N2 > N3 (*p* < 0.01), while at Day 15 and Day 30, a higher enrichment effect was observed at a higher dose; accumulation of 4-NP in the muscle at Day 7 and Day 15 was higher in N1 and N2 than in N3 (*p* < 0.01); while enrichment of 4-NP in gills was not highly correlated to its concentration. In the presence of MMT, enrichment of 4-NP in the liver was significantly decreased at 7 d in all N1 groups (*p* < 0.01), but such decreased accumulation in N1 was only observed in N1M1 at 15 d. Accumulation of 4-NP was enhanced in all N2 and N3 groups except for the N2M1 group (*p* < 0.01), but such enhanced accumulation was observed only in the N2M2 group until Day 30. In the muscle, enrichment of 4-NP at Day 7 was reduced by MMT in N1, but increased by MMT in N2 and N3, and the altered enrichment was maintained until Day 30 only in the N1M3 group. The concentration of 4-NP in gills was influenced by MMT and was reduced in all the experimental groups, but the difference was significant in only a few groups. In summary, as long as there was a significant difference, higher concentrations of MMT or 4-NP always led to higher accumulation of 4-NP when the other was constant. 

It can be speculated from the above-mentioned results that MMT exhibits different enrichment effects on 4-NP in zebrafish. MMT adsorbs 4-NP and reduces the actual concentration of 4-NP by flocculation in water, and this effect is directly reflected in the water-contacted gills, in which the short-term enrichment of 4-NP is reduced by MMT; once 4-NP is ingested by the zebrafish, MMT contributes to the short-term accumulation of 4-NP in the liver at both medium and high doses and in the muscle at a high dose, as well, but not to the accumulation of 4-NP in the liver and muscle at a low dose. Analysis of the experimental data also showed that the effects of MMT on 4-NP enrichment decrease gradually over time. Due to the limitation of this study that only three time points were designed for each experimental group, further research is required to identify the time points when the maximum concentration of 4-NP is achieved.

The enzyme activity of liver SOD and GST was affected by the organic 4-NP content. While the concentration of 4-NP is lower than 0.00747 μg/g, the activity of SOD would be induced. By contrast, the activity of SOD would be inhibited when the concentration of 4-NP is higher than 0.00747 μg/g, and the inductive and inhibiting effect would be increased with the increase of the concentration of 4-NP; the concentration of 4-NP had a similar effect on the enzyme activity of GST, while the critical concentration was 0.0401 μg/g. The induced enzyme activity of SOD could reach 175.82% compared to the control enzyme activity, while the GST could only reach 139.65% of the control enzyme activity. These results suggested that the SOD is more sensitive to the toxic effect of internal 4-NP, and 4-NP has a more effective regulatory mechanism on the enzyme activity of SOD.

## 5. Conclusions

According to our results, in the short term, MMT could possibly reduce the enrichment of nonylphenol in gills, and the high concentration of nonylphenol has the benefit of enriching itself in liver and muscle, while the low concentration of nonylphenol would be against its enrichment. The enzymatic activity of SOD and GST exerts an inductive effect when the concentration of nonylphenol in liver was reduced; by contrast, the enzymatic activity of SOD and GST would exert an inhibiting effect while a high-concentration of nonylphenol was gathered in the liver.

## Figures and Tables

**Table 1:** Average recovery rate of 4-NP.

Samples	Content (μg/L)	Average Recovery Rate % (*n* = 3)
Water	2.092 × 10^1^	77.797%
	2.092 × 10^2^	78.359%
	2.092 × 10^3^	89.274%
Liver	2.092 × 10^1^	78.539%
	2.092 × 10^2^	78.695%
	2.092 × 10^3^	81.126%
Muscle	2.092 × 10^1^	85.467%
	2.092 × 10^2^	79.503%
	2.092 × 10^3^	82.031%
Gill	2.092 × 10^1^	77.998%
	2.092 × 10^2^	79.235%
	2.092 × 10^3^	84.535%

**Table 2:** Regression equations and correlation coefficients of the standard curves.

Samples	Equations	Correlation Coefficient (R^2^)
Water	y = 370.65x + 431,635	0.9965
Liver	y = 362.77x + 356,899	0.9931
Muscle	y = 366.87x + 400,058	0.9922
Gill	y = 361.34x + 451,265	0.9909

**Table 3:** Statistical analysis of 4-NP contents in liver at Day 7.

	4-NP Contents (μg/g)	N2	N3	N1M1	N1M2	N1M3	N2M1	N2M2	N2M3	N3M1	N3M2	N3M3
N1	0.1224 ± 0.0096	**	**	**	**	**						
N2	0.0714 ± 0.0066		/				/	**	**			
N3	0.0711 ± 0.0014									**	**	**
N1M1	0.0699 ± 0.0099				*	**	/			/		
N1M2	0.085 ± 0.0129					*		*			/	
N1M3	0.099 ± 0.0073								/			/
N2M1	0.0747 ± 0.006							**	**	/		
N2M2	0.1001 ± 0.008								/		**	
N2M3	0.1021 ± 0.0121											/
N3M1	0.0787 ± 0.0035										/	*
N3M2	0.0834 ± 0.0066											/
N3M3	0.0912 ± 0.0091											

**Table 4:** Statistical analysis of 4-NP contents in liver at Day 15.

	4-NP Contents (μg/g)	N2	N3	N1M1	N1M2	N1M3	N2M1	N2M2	N2M3	N3M1	N3M2	N3M3
N1	0.0347 ± 0.0039	**	**	**	/	/						
N2	0.0422 ± 0.002		**				**	**	/			
N3	0.0517 ± 0.0022									**	/	**
N1M1	0.0231 ± 0.0068				**	**	**			*		
N1M2	0.0358 ± 0.0029					*		/			/	
N1M3	0.0401 ± 0.0021								/			*
N2M1	0.0253 ± 0.0066							*	**	*		
N2M2	0.0346 ± 0.0053								*		/	
N2M3	0.0425 ± 0.0033											**
N3M1	0.0338 ± 0.0042										/	**
N3M2	0.0349 ± 0.004											**
N3M3	0.0465 ± 0.0067											

**Table 5:** Statistical analysis of 4-NP contents in liver at Day 30.

	4-NP Contents (μg/g)	N2	N3	N1M1	N1M3	N2M1	N2M2	N2M3	N3M1	N3M2	N3M3
N1	0.0331 ± 0.0038	/	*	/	/						
N2	0.0318 ± 0.0035		*			/	*	*			
N3	0.0408 ± 0.0063								/	/	/
N1M1	0.0357 ± 0.0045				/	/			/		
N1M2	0.0341 ± 0.004				/		/			/	
N1M3	0.0344 ± 0.0055							/			*
N2M1	0.0354 ± 0.0034						/	/	/		
N2M2	0.0389 ± 0.0062							/		/	
N2M3	0.0366 ± 0.0040										*
N3M1	0.0325 ± 0.0077									/	*
N3M2	0.0368 ± 0.0036										*
N3M3	0.0436 ± 0.0046										

**Table 6:** Statistical analysis of 4-NP contents in muscle at Day 7.

	4-NP Contents (μg/g)	N2	N3	N1M1	N1M2	N1M3	N2M1	N2M2	N2M3	N3M1	N3M2	N3M3
N1	0.0894 ± 0.0062	/	**	**	**	*						
N2	0.0976 ± 0.0118		**				/	/	/			
N3	0.0696 ± 0.0072									**	**	**
N1M1	0.0620 ± 0.0028				/	**	**			**		
N1M2	0.0649 ± 0.0020					**		**			**	
N1M3	0.0776 ± 0.0070								**			*
N2M1	0.0909 ± 0.0022							**	*	*		
N2M2	0.1054 ± 0.0091								/		/	
N2M3	0.1039 ± 0.0097											/
N3M1	0.1062 ± 0.0125										/	/
N3M2	0.0888 ± 0.0113											/
N3M3	0.0971 ± 0.014											

**Table 7:** Statistical analysis of 4-NP contents in muscle at Day 15.

	4-NP Contents (μg/g)	N2	N3	N1M1	N1M2	N1M3	N2M1	N2M2	N2M3	N3M1	N3M2	N3M3
N1	0.0390 ± 0.0022	*	**	/	/	*						
N2	0.0430 ± 0.0033		**				/	/	/			
N3	0.0348 ± 0.0015									/	/	/
N1M1	0.0363 ± 0.0046				/	/	/			/		
N1M2	0.0332 ± 0.0071					/		/			/	
N1M3	0.0349 ± 0.0036								/			/
N2M1	0.0384 ± 0.0048							/	/	/		
N2M2	0.0388 ± 0.0034								/		/	
N2M3	0.0383 ± 0.0073											/
N3M1	0.0365 ± 0.0060										/	/
N3M2	0.0426 ± 0.0086											/
N3M3	0.038 ± 0.0085											

**Table 8:** Statistical analysis of 4-NP contents in muscle at Day 30.

	4-NP Contents (μg/g)	N2	N3	N1M1	N1M2	N1M3	N2M1	N2M2	N2M3	N3M1	N3M2	N3M3
N1	0.0410 ± 0.0065	/	/	*	/	*						
N2	0.0399 ± 0.0057		/				/	/	/			
N3	0.0333 ± 0.0114									/	/	/
N1M1	0.0263 ± 0.0121				/	/	/			/		
N1M2	0.0357 ± 0.0073					/		/			/	
N1M3	0.0328 ± 0.0048								/			/
N2M1	0.0333 ± 0.0101							/	/	/		
N2M2	0.0448 ± 0.0117								/		/	
N2M3	0.0348 ± 0.0075											/
N3M1	0.0373 ± 0.0063										/	/
N3M2	0.0480 ± 0.0130											/
N3M3	0.0361 ± 0.003											

**Table 9:** Statistical analysis of 4-NP contents in gill at Day 7.

	4-NP Contents (μg/g)	N2	N3	N1M1	N1M2	N1M3	N2M1	N2M2	N2M3	N3M1	N3M2	N3M3
N1	0.0988 ± 0.007	/	**	**	**	**						
N2	0.1090 ± 0.0195		/				*	**	/			
N3	0.1281 ± 0.0122									*	/	/
N1M1	0.0687 ± 0.0046				/	/	*			**		
N1M2	0.0759 ± 0.0104					/		/			/	
N1M3	0.0759 ± 0.0089								**			*
N2M1	0.0833 ± 0.0103							/	/	/		
N2M2	0.0824 ± 0.0042								**		/	
N2M3	0.0932 ± 0.0061											/
N3M1	0.0885 ± 0.0075										/	/
N3M2	0.0817 ± 0.0111											/
N3M3	0.0859 ± 0.0062											

**Table 10:** Statistical analysis of 4-NP contents in gill at Day 15.

	4-NP Contents (μg/g)	N2	N3	N1M1	N1M2	N1M3	N2M1	N2M2	N2M3	N3M1	N3M2	N3M3
N1	0.0425 ± 0.0072	/	/	*	/	*						
N2	0.0363 ± 0.0068		/				/	/	/			
N3	0.0398 ± 0.0029									/	/	/
N1M1	0.0328 ± 0.0065				/	/	/			/		
N1M2	0.0410 ± 0.0079					/		/			/	
N1M3	0.0316 ± 0.0095								/			/
N2M1	0.0361 ± 0.0058							/	/	/		
N2M2	0.0425 ± 0.0049								/		/	
N2M3	0.0381 ± 0.0072											/
N3M1	0.0334 ± 0.0048										/	/
N3M2	0.0378 ± 0.0051											/
N3M3	0.0392 ± 0.0054											

**Table 11:** Statistical analysis of 4-NP contents in gill at Day 30.

	4-NP Contents (μg/g)	N2	N3	N1M1	N1M2	N1M3	N2M1	N2M2	N2M3	N3M1	N3M2	N3M3
N1	0.0414 ± 0.0048	/	/	*	*	/						
N2	0.0398 ± 0.0098		/				/	/	/			
N3	0.0430 ± 0.0036									*	/	/
N1M1	0.0309 ± 0.0086				/	*	/			/		
N1M2	0.0302 ± 0.0099					/		/			/	
N1M3	0.0400 ± 0.0052								/			/
N2M1	0.0308 ± 0.0054							/	*	*		
N2M2	0.0398 ± 0.0103								/		/	
N2M3	0.0374 ± 0.0035											/
N3M1	0.0378 ± 0.0034										/	/
N3M2	0.0385 ± 0.0057											/
N3M3	0.0406 ± 0.0072											
